# Artificial intelligence-quantified fundus tessellated density and its association with myopia severity in Chinese children: a cross-sectional study

**DOI:** 10.3389/fcell.2026.1754792

**Published:** 2026-07-08

**Authors:** Nan Liu, Zhaosheng Li, Xue Zhang, Saiguang Ling, Jifeng Yu, Li Li

**Affiliations:** 1 Department of Ophthalmology, Key Laboratory of Major Diseases in Children, National Center for Children’s Health, Ministry of Education, Beijing Children’s Hospital Capital Medical University, Beijing, China; 2 Department of Ophthalmology, Shunyi Women's and Children's Hospital of Beijing Children's Hospital, Beijing, China; 3 EVision Technology (Beijing) Co. LTD., Beijing, China

**Keywords:** artificial intelligence, axial length, fundus tessellation, high myopia, receiver operating characteristic curve

## Abstract

**Introduction:**

To quantitatively assess the whole fundus tessellated density (FTD) using artificial intelligence (AI) and evaluate its association with myopia severity in Chinese children with myopia.

**Methods:**

A cross-sectional analysis was conducted on participants with myopia at Beijing Children’s Hospital Capital Medical University, from October 2023 to June 2024. AI technology was used to quantitatively measure FTD based on fundus photographs. Participants were classified into mild, moderate, and high myopia (HM) groups based on cycloplegic spherical equivalent (SE). To explore the relationship between FTD and SE, Spearman correlation coefficients along with linear regression analysis were utilized. The ability of FTD to discriminate HM was assessed using Receiver Operating Characteristic curve.

**Results:**

The study included 263 eyes from children aged 6–12 years. AI-quantified FTD showed excellent agreement with manual grading of fundus tessellation (area under the curve [AUC] = 0.97, 95% confidence interval [CI]: 0.95–0.98). Median FTD values were 0.01 (0.00–0.02), 0.01 (0.00–0.03), and 0.04 (0.01–0.12) for the three groups respectively. Significant difference was found when comparing HM group with the mild and moderate myopia groups (both *P* < 0.05). A negative correlation between FTD and SE was observed (*ρ* = −0.41, *P* < 0.001). After adjusting for age and sex, multiple linear regression analysis revealed that each 0.01 increase in FTD was associated with a 0.07 D reduction in SE (β = −7.17 per 1.00 FTD, equivalent to −0.07 per 0.01 FTD, 95% CI: −0.10 to −0.05, *P* < 0.001). The AUC of FTD for identifing HM was 0.75 (95% CI: 0.68–0.81). Combining FTD with axial length-to-corneal radius ratio (AL/CR) improved the discriminative performance, achieving an AUC of 0.94 (95% CI: 0.91–0.97), significantly higher than axial length (AL), AL/CR, FTD, or FTD combined with AL (all *P* < 0.05).

**Conclusion:**

AI -quantified FTD demonstrates a significant correlation with the severity of myopia.

## 1 Introduction

Myopia represents the most prevalent form of refractive error worldwide and has escalated into a growing epidemic of high myopia (HM), largely attributable to the declining age of onset and increasingly rapid progression observed in recent decades (Morgan et al., 2018; Wu et al., 2016). By 2050, HM is expected to affect approximately 938 million people worldwide (Wong and Saw, 2016). As myopia progresses, the risk of structural damage to ocular tissues increases significantly, particularly in HM cases (Baird et al., 2020). HM is strongly associated with vision-threatening complications such as cataract, myopic macular degeneration, and glaucoma (Fan et al., 2024). Identifying reliable biomarkers for HM is therefore essential for early monitoring, lifestyle-based interventions, and strategies to slow myopia progression (Han et al., 2022; Tideman et al., 2019; Wang et al., 2021).

Fundus tessellation (FT) is characterized by visible choroidal vessels in the posterior segment of the eye extending beyond the peripapillary area, and increased FT is considered a key biomarker associated with the risk of developing both myopia and HM (Gong et al., 2024; Tian et al., 2024; Yan et al., 2015). Advances in artificial intelligence (AI) and image processing now enable the precise diagnosis of FT by objectively quantifying it as fundus tessellated density (FTD), a significant improvement over inaccurate traditional methods (Gong et al., 2024; Shao et al., 2021; Song et al., 2018; Yan et al., 2015). A recent comprehensive review has summarized these methodological advancements and highlighted the potential of AI-derived FTD as a quantitative imaging biomarker for myopic structural changes (Guo et al., 2026). Recent longitudinal evidence further supports that FTD increases over time in adults with HM and correlates closely with axial elongation, suggesting its potential as a quantitative biomarker for monitoring myopia progression (Ten et al., 2026). Current researches on the prevalence of FTD and its association with myopia have primarily focused on adult or mixed populations with varying refractive status (Huang et al., 2023a; Shao et al., 2021; Wei et al., 2024; Xiao et al., 2026). However, studies specifically targeting populations with myopia remain limited, particularly children, a key group for myopia control and prevention.

In light of this, we focused specifically on Chinese children with myopia to apply and validate an AI-based method for automated quantification of FTD. The clinical utility of AI-quantified FTD was systematically evaluated within this population. Furthermore, for the first time, we comprehensively investigated whether integrating FTD with established biometric parameters (axial length, AL; axial length-to-corneal radius ratio, AL/CR) could produce a synergistic effect superior to using any single metric alone.

## 2 Methods

### 2.1 Participants

This cross-sectional, hospital-based study included children with myopia who visited the clinic at Beijing Children’s Hospital Capital Medical University, between October 2023 and June 2024. Myopia was defined as a cycloplegic spherical equivalent (SE) of −0.50 D or lower (Wei et al., 2024). The standards for inclusion were specified as follows: (1) children aged 6–12 years; (2) first diagnosis of myopia at this hospital; (3) parental or guardian consent for AI testing; and (4) fundus photography conducted within 1 month after cycloplegic refraction. The criteria for exclusion were outlined as follows: (1) patients with other eye diseases such as strabismus, amblyopia, glaucoma, etc.,; (2) systemic conditions associated with HM, including but not limited to Marfan syndrome and Stickler syndrome; (3) history of ocular surgery; (4) prior use of any myopia control interventions; and (5) poor quality of fundus images. Based on cycloplegic SE, participants were classified into the following three groups: mild myopia (≤−0.50 D and > −3.00 D), moderate myopia (≤−3.00 D and > −6.00 D), and HM group (≤−6.00 D) (Ma et al., 2016; Ojaimi et al., 2005). The study received ethical approval from the Ethics Committee of Beijing Children’s Hospital (No [2023]-E−140-Y) and was conducted in accordance with the Declaration of Helsinki. Oral informed consent was obtained from the parents or legal guardians of all participants.

Sample size was calculated using PASS 2021 software for three independent group means. The power was set at 80% (1-β = 0.80), with a significance level of 0.05 (α = 0.05). A total of three groups (k = 3) with a 1:1:1 allocation ratio were assumed. Based on our preliminary data, the mean SE values for the mild, moderate, and HM groups were-1.72 D, −4.25 D, and −7.82 D, respectively, with corresponding standard deviations of 0.58, 0.93, and 1.74. These preliminary data were derived from a pilot study conducted at our hospital before the main study. The calculated required sample size was 72 participants per group, totaling 216 participants. Accounting for a 10% dropout rate, each group required at least 80 participants, resulting in a minimum total sample size of 240 participants. Our final sample of 263 participants (82 with mild myopia, 92 with moderate myopia, and 89 with high myopia) exceeded this requirement, confirming adequate statistical power for the main analyses.

### 2.2 Refractive measurement

Autorefraction was conducted using an automatic computer keratometer (KR-800, Topcon, Japan). Cycloplegic refraction was measured after administering compound tropicamide eye drops (SINQI) three times at 10-min intervals. After 30 min, cycloplegia was diagnosed through an assessment of the pupillary light reflex, characterized by the lack of this reflex and a pupil diameter exceeding 6 mm. If the reflex persisted after 30 min, additional drops were applied until the reflex disappeared. A trained optometrist then measured static refraction. Three readings were taken, and the average was calculated. If any two values differed by more than 0.50 D, the measurement was repeated. The SE was calculated as the sum of the spherical power and half of the cylindrical power.

### 2.3 Assessment of ocular biological metrics

AL and corneal curvature were measured under non-cycloplegic conditions using an optical interference AL measurement device (AL-Scan, NIDEK, Japan). Five consecutive measurements were taken and automatically averaged. To ensure uniform tear film coverage, participants were instructed to blink before each assessment. A measurement was considered valid if the variation among readings was less than 0.02 mm. All the above examinations were performed by the same examiner, and the measurement time was 09:00–10:00 am. Mean corneal curvature was calculated as the average of the maximum and minimum curvature values. The corneal radius (CR) was determined using the formula: CR=1000×0.3375/mean corneal curvature . AL/CR was calculated by performing a division of AL by CR (He et al., 2015).

### 2.4 Quantitative analysis of FTD

**FIGURE 1:**
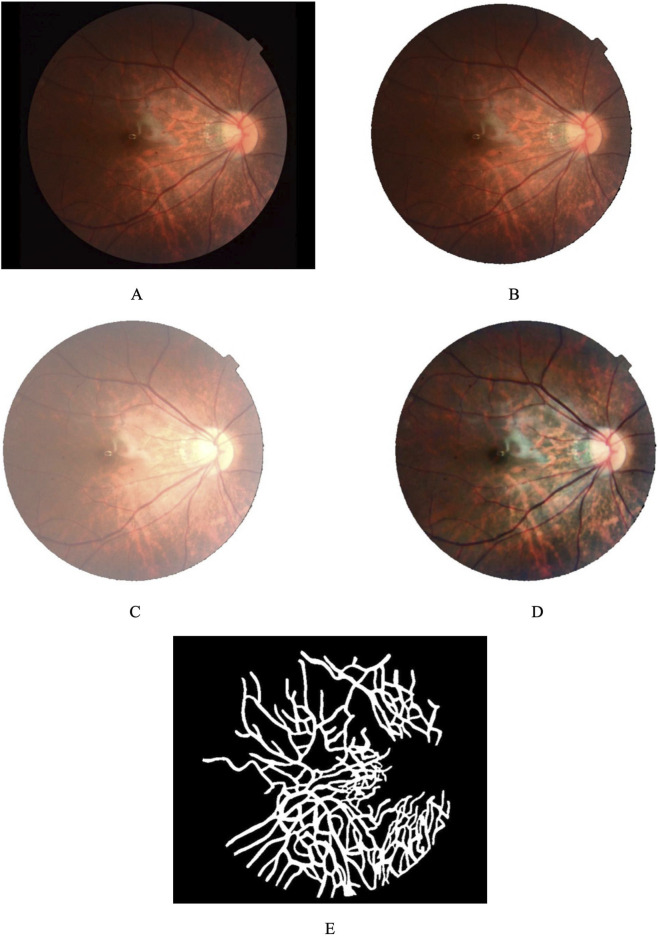
Workflow for extracting FTD using AI **(A)** Original fundus image **(B)** Region of interest **(C)** Image after denoising and normalization **(D)** Enhanced image highlighting internal structures **(E)** Annotated image showing exposed choroid based on the enhanced image. FTD, fundus tessellated density; AI, artificial intelligence.

The fundus was photographed with a camera designed for non-mydriatic imaging (TRC-NW400, Topcon, Tokyo, Japan). All fundus images were acquired using 45° macular-centered fields. Two trained ophthalmologists (each with more than 3 years of experience in retinal image interpretation) independently graded fundus tessellation in a blinded manner. Inter-observer agreement was assessed using Cohen’s kappa statistics, yielding κ > 0.80, indicating excellent agreement. Intra-observer consistency was evaluated by regrading 100 images after a two-week interval, also yielding κ > 0.80. Discrepancies were resolved by a senior retinal specialist (Huang et al., 2023b). The quantitative assessment of FTD was conducted using intelligent analysis software tailored for color fundus photography [EVisionAI, EVision Technology (Beijing) Co. Ltd.] (Wang et al., 2022). The mean area of visible choroid relative to each unit area of the fundus was calculated and defined as FTD (Huang et al., 2023b; Li et al., 2023; Shao et al., 2021). As shown in Figure 1, the FTD calculation process involved image preprocessing, sample labeling, deep learning (DL) segmentation, and final computation.

Image preprocessing included the following four steps to ensure consistent image quality and reduce inter-device variability: region of interest extraction via red-channel thresholding and morphological analysis; denoising using frequency-domain low-pass filtering; normalization of brightness and color in LAB space; contrast enhancement with CLAHE (Xu et al., 2019). A camera-agnostic method was used for pixel pitch calibration based on automated region of interest and optic disc detection. This method derived a stable region of interest–disc diameter ratio (6.40 ± 0.62 pixels) from 40,600 fundus images of 45° FOV, enabling accurate pixel pitch estimation with less than 5% error compared with ISO-standard manual measurements (Long et al., 2022). This allows quantitative assessment of fundus structures without prior knowledge of camera parameters. Tessellation grading followed a standardized protocol wherein graders accounted for image contrast, brightness, background pigmentation, and used reference standards for calibration. The use of images from multiple cameras and preprocessing steps helped mitigate the effect of fundus pigmentation (He et al., 2023).

In the labeling stage, a semi-automated method combining automatic labeling and manual correction was used. Automatic labeling was performed through channel separation and subtraction of color fundus images. The resulting samples were then manually corrected to produce the final sample image.

In the stage of DL segmentation, a semantic segmentation model based on DL (TransUNet) was then employed to extract the exposed choroid. The TransUNet architecture integrates a Vision Transformer encoder with a convolutional neural network backbone to capture both local features and long-range global dependencies. The decoder employs progressive upsampling with skip connections to restore spatial resolution and refine segmentation boundaries. Pixel-level predictions are obtained by thresholding the confidence probability map; the optimal threshold was empirically determined on the validation set by maximizing the Dice coefficient. The dataset comprised 1,160 images with tessellation and 2,780 images with patchy chorioretinal atrophy, each independently annotated by two retinal specialists. The annotation process followed a rigorous two-step procedure: initial delineation by one ophthalmologist, followed by thorough review and correction by a second specialist to ensure label consistency and reliability. The dataset was partitioned into training and testing sets at a 7:3 ratio. Performance metrics evaluated at the pixel level demonstrated high accuracy, with tessellation detection achieving an accuracy of 0.98, sensitivity of 0.96, and specificity of 0.98; corresponding values for patchy atrophy were 0.99, 0.98, and 0.99, respectively (He et al., 2023). In addition to these pixel-level metrics, we recognize the importance of overlap-based metrics for independent model validation. For example, in a curriculum learning-based fully automated system for choroidal structure quantification in highly myopic patients, Liu et al. achieved a Dice Similarity Coefficient of 0.9221 and an Intersection over Union of 0.8575 (Liu et al., 2022). Although our current study did not originally compute Dice Similarity Coefficient and Intersection over Union, we are actively evaluating these metrics as part of ongoing validation efforts and will report them in future updates.

Finally, FTD was calculated by determining the average exposed choroidal area per unit area of the fundus (Shao et al., 2021).

To facilitate reproducibility, we provide the following additional details: The encoder of TransUNet uses a Vision Transformer to capture global contextual features. The decoder employs progressive upsampling with skip connections to restore spatial resolution. Pixel-level predictions were obtained via thresholding of confidence probability maps.

### 2.5 Agreement between manual grading and automated FTD quantification

To evaluate the agreement between manual grading and automated FTD quantification, we compared the AI-derived FTD values with manual binary grading of FT (presence vs. absence of FT). Manual binary grading served as the reference standard. The manual grading procedure (two trained ophthalmologists, κ > 0.80 for both inter-observer and intra-observer agreement, discrepancies resolved by a senior retinal specialist) is detailed in Section 2.4. (Liu et al., 2022). During grading, image parameters such as contrast, brightness, background pigmentation, and overall image quality were considered, and standard reference images were used to minimize subjective bias.

Based on the grading system described by Yan et al. (2015), the whole FT was classified into four grades: grade 0 (no visible choroidal vessels outside the parapapillary beta zone), grade 1 (slight visibility), grade 2 (moderate visibility), and grade 3 (marked visibility). For the purpose of binary classification in this validation analysis, grades ≥1 were defined as the presence of FT, while grade 0 was defined as the absence of FT.

### 2.6 Statistical analysis

The analysis and visualization of data were performed with R 4.1.1 software. Quantitative variables with normal distributions were presented as mean (SD), and group comparisons were performed using one-way ANOVA, followed by Tukey’s HSD test for pairwise comparisons. Non-normally distributed quantitative variables were presented as median (P_25_, P_75_). Group comparisons were made with the Kruskal–Wallis test, followed by Bonferroni-corrected pairwise tests. The qualitative results were presented in absolute terms (percentages), and Fisher’s Exact test was utilized to analyze the differences among the groups. Spearman correlation analysis was used for the initial exploration of associations between variables. Subsequently, a multiple linear regression model, adjusted for potential confounders including age and sex, was employed to further investigate the quantitative relationship between FTD and SE. The discriminative value of FTD for HM was evaluated using Receiver Operating Characteristic (ROC) curve analysis. Differences between area under the curves (AUCs) were assessed using the DeLong test. Spearman correlation analysis and ROC curve analysis were also used to assess the agreement between manual grading and automated FTD quantification. A two-sided *P* < 0.05 was considered to indicate statistical significance.

## 3 Results

### 3.1 General characteristics

**FIGURE 2:**
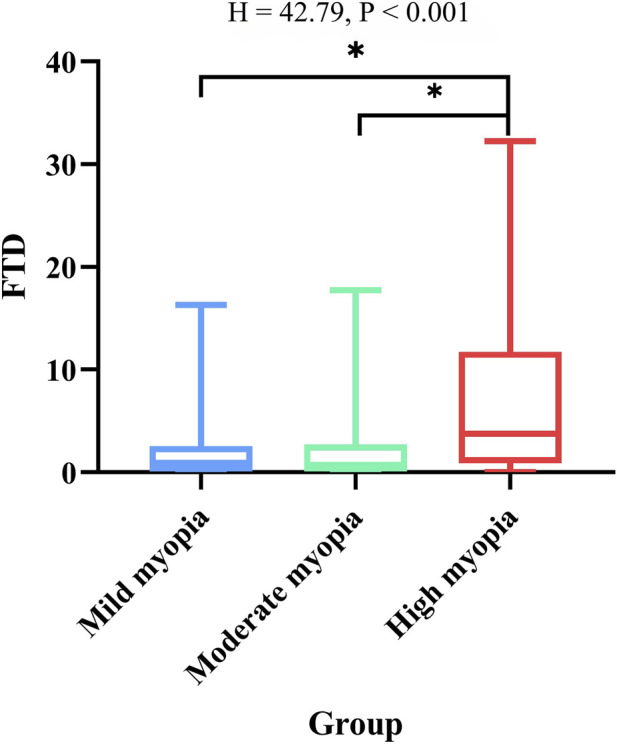
Box plot of FTD distribution by myopia severity. FTD, fundus tessellated density.

Due to the strong correlation observed between both eyes, this research concentrated solely on data obtained from the right eye (Thorn et al., 2020). A total of 263 eyes from 263 participants with myopia were analyzed, with a mean age of 9.84 ± 1.58 years. The numbers of participants in the mild, moderate, and HM groups were 82, 92, and 89, respectively. The median FTD values across the three groups were 0.01 (0.00–0.02), 0.01 (0.00–0.03), and 0.04 (0.01–0.12), respectively. Statistical analysis revealed a significant difference between HM group and both mild and moderate groups (both *P* < 0.05), whereas no statistically significant difference was observed between mild and moderate groups (*P* = 0.949). Detailed characteristics of all parameters are presented in Table 1, and the distribution of FTD across groups is illustrated in Figure 2.

**TABLE 1:** General characteristics of participants.

Variables	Mild myopia (N = 82)	Moderate myopia (N = 92)	High myopia (N = 89)	F/H	*P*
Age (year), median (P_25_, P_75_)	9 (8, 11)	10 (9, 11)	10 (8, 11)	5.64^†^	0.060
Male, no (%)	47 (57.32%)	47 (51.09%)	51 (57.30%)	-	0.634
SE(D), median (P_25_, P_75_)	−1.57 (−2.03, −1.13)	−4.13 (−4.84, −3.50)^a^	−7.00 (−8.31, −6.38)^a^ ^,^ ^b^	232.80^†^	<0.001
AL (mm), mean ± SD	24.00 ± 0.74	25.05 ± 0.88^a^	26.16 ± 0.95^a^ ^,^ ^b^	133.67*	<0.001
CR, mean ± SD	7.78 ± 0.24	7.75 ± 0.26	7.76 ± 0.29	0.266*	0.767
AL/CR, median (P_25_, P_75_)	3.08 (3.05, 3.12)	3.24 (3.19, 3.30)^a^	3.3 7 (3.30, 3.44)^a^ ^,^ ^b^	169.99^†^	<0.001
FTD (%), median (P_25_, P_75_)	0.01 (0.00, 0.02)	0.01 (0.00, 0.03)	0.04 (0.01, 0.12)^a^ ^,^ ^b^	42.79^†^	<0.001

SE, spherical equivalent; AL, axial length; CR, corneal radius; AL/CR, axial length to corneal radius ratio; FTD, fundus tessellated density; SD, standard deviation.

^a^ *P* < 0.05 compared with mild myopia.

^b^ *P* < 0.05 compared with moderate myopia

* F value of one-way ANOVA.

^†^ H value of KW H test.

### 3.2 Agreement between manual grading and automated FTD quantification

**FIGURE 3:**
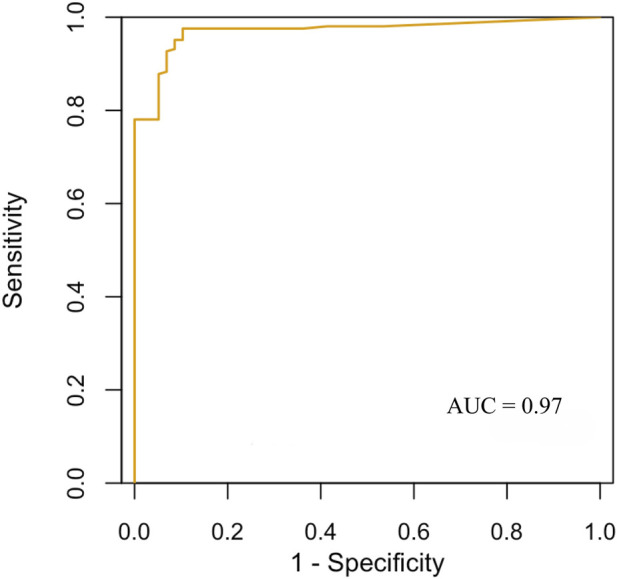
ROC curve of AI-quantified FTD for identifying FT. ROC, Receiver Operating Characteristic; AI, artificial intelligence; FTD, fundus tessellated density; FT, fundus tessellation.

Spearman correlation analysis revealed a strong positive correlation between AI-quantified FTD and manual binary grading of FT, with a correlation coefficient of 0.68 (95% CI: 0.60 to 0.74, *P* < 0.001). ROC analysis further demonstrated excellent agreement between AI-quantified FTD and manual grading. The AUC was 0.97 (95% CI: 0.95–0.98). The optimal FTD cutoff for detecting the presence of FT was 0.02, yielding a sensitivity of 0.98, specificity of 0.90, positive predictive value (PPV) of 0.97, and negative predictive value (NPV) of 0.91 (Figure 3).

### 3.3 Spearman correlation analysis

**FIGURE 4:**
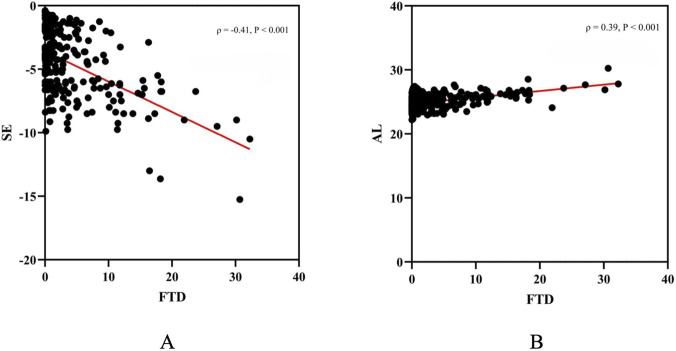
Scatter plots of correlations between FTD and ocular parameters **(A)** FTD and SE **(B)** FTD and AL. FTD, fundus tessellated density; SE, Spherical Equivalent; AL, axial length.

The associations between SE, ocular parameters, and FTD are presented in Table 2. A moderate negative correlation was observed between FTD and SE, with a correlation coefficient of −0.41 (95% CI: −0.51 to −0.30, *P* < 0.001). FTD was significantly positively correlated with both AL and AL/CR, with correlation coefficients of 0.39 (95% CI: 0.28 to 0.50, *P* < 0.001) and 0.29 (95% CI: 0.17 to 0.40, *P* < 0.001), respectively. Although the correlation between FTD and CR was statistically significant (*P* = 0.013), the strength of the association was weak, with correlation coefficients of 0.15 (95% CI: 0.03–0.28). Scatter plots illustrating the correlations between FTD and SE, and between FTD and AL, are shown in Figure 4.

**TABLE 2:** Association between ocular parameters, SE and FTD.

Variables	Correlation coefficients	95% CI	*P*
AL and SE	*ρ* = −0.77	−0.81 to −0.70	<0.001
CR and SE	*ρ* = 0.06	−0.06 to 0.18	0.327
AL/CR and SE	*ρ* = −0.86	−0.89 to −0.82	<0.001
FTD and SE	*ρ* = −0.41	−0.51 to −0.30	<0.001
FTD and AL	*ρ* = 0.39	0.28 to 0.50	<0.001
FTD and CR	*ρ* = 0.15	0.03 to 0.28	0.013
FTD and AL/CR	*ρ* = 0.29	0.17 to 0.40	<0.001

SE, spherical equivalent; AL, axial length; CR, corneal radius; AL/CR, axial length to corneal radius ratio; FTD.

fundus tessellated density; CI, confidence interval.

### 3.4 Multiple linear regression analysis

As detailed in Table 3, multiple linear regression analysis confirmed that FTD was significantly and independently associated with SE in both the unadjusted model (Model 1: β = −9.07, 95% CI: −11.90 to −6.30, *P* < 0.001) and the model adjusted for age and sex (Model 2: β = −7.17, 95% CI: −9.80 to −4.50, *P* < 0.001). Given that the numerical range of FTD values was small in our sample, a “one-unit increase” is not clinically meaningful. After rescaling, each 0.01 increase in FTD was associated with a 0.07 D reduction in SE (β = −7.17 per 1.00 FTD, equivalent to −0.07 per 0.01 FTD, 95% CI: −0.10 to −0.05, *P* < 0.001) in the adjusted model. In Model 2, both age (β = 0.26, 95% CI: 0.17 to 0.35, *P* < 0.001) and sex (β = 0.51, 95% CI: 0.24 to 0.80, *P* < 0.001) were also significantly associated with SE.

**TABLE 3:** Multivariable linear regression analysis of variables with SE.

Variables	Model 1 (unadjusted)				Model 2 (Adjusted for age and gender)			
	β(95% CI)	S.E.	t	*P*	β(95% CI)	S.E.	t	*P*
AL (mm)	−0.45 (-0.63 to −0.26)	0.09	−4.72	<0.001	−0.63 (-0.82 to −0.45)	0.09	−6.89	<0.001
AL/CR	−11.18 (-12.62 to −9.74)	0.73	−15.28	<0.001	−10.82 (-12.21 to −9.52)	0.68	−15.90	<0.001
FTD	−9.07 (-11.90 to −6.30)	1.43	−6.34	<0.001	−7.17 (-9.80 to −4.50)	1.34	−5.34	<0.001
Age (years)	-	-	-	-	0.26 (0.17–0.35)	0.04	5.89	<0.001
Gender (ref: male)	-	-	-	-	0.51 (0.24–0.80)	0.14	3.67	<0.001

AL, axial length; AL/CR, axial length to corneal radius ratio; FTD, fundus tessellated density; β, unstandardized beta; S.E., standard error; SE, spherical equivalent.

### 3.5 ROC curve analysis for HM identification

**FIGURE 5:**
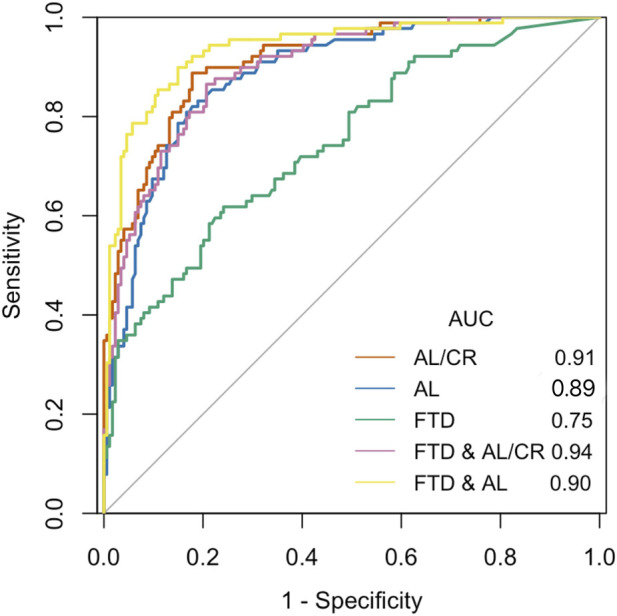
ROC curves of different parameters for identifying HM. ROC, Receiver Operating Characteristic; HM, high myopia.

As shown in Table 4, the AUCs for FTD, AL, AL/CR were 0.75 (95% CI: 0.68–0.81), 0.89 (95% CI: 0.85–0.93), and 0.91 (95% CI:0.88–0.95), respectively. The optimal FTD cutoff for identifying HM was 0.03, yielding a sensitivity of 0.62, specificity of 0.76, PPV of 0.57, and NPV of 0.80. The DeLong test revealed significant differences between FTD and both AL and AL/CR (both *P* < 0.05), while no significant difference was found between AL and AL/CR (P = 0.260). When FTD was combined with AL, the AUC increased to 0.90 (95% CI: 0.86–0.94), representing a significant improvement over FTD alone (*P* < 0.001), but not over AL (*P* = 0.181) or AL/CR (*P* = 0.532). Combining FTD with AL/CR further increased the AUC to 0.94 (95% CI: 0.91–0.97), with a sensitivity of 0.90, specificity of 0.85, PPV of 0.75, and NPV of 0.96. This combined model demonstrated statistically significant improvement in AUC compared with all other variables (all *P* < 0.05). Table 4 presents the AUC, sensitivity, and specificity of all variables for HM identification, and Figure 5 illustrates the ROC curve comparisons of all variables for HM identification.

**TABLE 4:** ROC curve analysis of all variables.

Variables	AUC(95% CI)	Sensitivity	Specificity	Youden index	Cutoff	*P*	PPV	NPV
AL	0.89 (0.85–0.93)	0.81	0.83	0.64	25.51	<0.001	0.71	0.90
AL/CR	0.91 (0.88–0.95)	0.89	0.82	0.71	3.27	<0.001	0.71	0.93
FTD^†^	0.75 (0.68–0.81)	0.62	0.76	0.38	0.03	<0.001	0.57	0.80
FTD and AL	0.90 (0.86–0.94)	0.87	0.80	0.66	0.28	<0.001	0.68	0.92
FTD and AL/CR*	0.94 (0.91–0.97)	0.90	0.85	0.75	0.32	<0.001	0.75	0.96

AL, axial length; AL/CR, axial length to corneal radius ratio; FTD, fundus tessellated density; ROC, receiver operating characteristic; AUC, area under the curve; CI, confidence interval; PPV, positive predictive value; NPV, negative predictive value.

^†^ Comparison of the AUCs, between FTD, and AL,AL/CR, and FTD, combined with AL, using Delong test (all *P* < 0.05).

* Comparison of the AUCs, between FTD, combined with AL/CR, and AL,AL/CR,FTD, and FTD, combined with AL, using Delong test (all *P* < 0.05).

## 4 Discussion

In this study, we applied an AI-based approach to quantitatively assess FTD in a cohort of Chinese children with myopia, and systematically evaluated its association with the severity of myopia. We further validated the agreement between AI-quantified FTD and manual binary grading of FT. Spearman correlation analysis revealed a strong positive correlation between AI-quantified FTD and manual grading (*ρ* = 0.68, 95% CI: 0.60 to 0.74, *P* < 0.001). ROC analysis demonstrated excellent discriminative ability of AI-quantified FTD for detecting the presence of FT, with an AUC of 0.97 (95% CI: 0.95–0.98) and an optimal cutoff of 0.02. These findings confirm the validity of our automated quantification approach. Notably, our results are highly consistent with the Nanjing Eye Study (Huang et al., 2023b). In their general pediatric population, the AUC for detecting grade ≥1 F T was 0.949 with a cutoff of 0.016, closely matching our findings (AUC = 0.97, cutoff = 0.02). Besides, Gong et al. previously reported that a FTD of 0.02 or higher in school-age children could indicate a tessellated fundus (Gong et al., 2024). The utility of DL-based FTD quantification has also been validated in longitudinal settings, with good reproducibility for detecting regional (macular and peripapillary) FTD changes over 5 years (Ten et al., 2026). These findings across different studies all support the robustness and generalizability of AI-based FTD quantification.

Prior studies have shown a consistent association between FT or FTD and the progression of myopia (Chen et al., 2023; Gong et al., 2024; Shao et al., 2021; Wei et al., 2024). However, the distribution of FTD across varying degrees of myopia remains unexplored, particularly in children. In our study, the median FTD values were 0.01 (0.00–0.02) in the mild myopia group, 0.01 (0.00–0.03) in the moderate myopia group, and 0.04 (0.01–0.12) in the HM group. These data bridge a critical gap in the existing knowledge by elucidating the FTD distribution profile in pediatric populations with myopia. Individuals with HM exhibited significantly higher FTD than those with mild and moderate myopia (both *P* < 0.05). This observed association may be primarily related to axial elongation mechanism underlying changes of FT. Axial elongation leads to thinning of large and medium choroidal vessels, as well as the surrounding intervascular tissue, altering the choroidal structure. As a result, the large choroidal vessels become more visible during ophthalmoscopic examination, leading to an increase in FT (Wei et al., 2024; Zhao et al., 2018). A recent pediatric study further revealed a nonlinear threshold effect: when subfoveal choroidal thickness falls below approximately 149 μm, FTD increases at a significantly accelerated rate (Zhang et al., 2026), suggesting a potential biomechanical tipping point in choroidal remodeling during myopia progression. However, the difference of FTD between the mild and moderate myopia groups was not significant (*P* = 0.949). This observation may be attributed to the multifactorial nature of FT, which involves various factors such as choroidal thickness, atrophy of the choriocapillaris, thinning of the retina and sclera, as well as systemic factors including age, sex, and body mass index (Chen et al., 2023; Huang et al., 2023a; Shao et al., 2021; Yamashita et al., 2020; Yan et al., 2015). Therefore, the significant increase of FTD prevalence in HM group highlights its cross-sectional association with HM.

Published evidence indicates that higher FTD was associated with longer AL and more myopic SE (Li et al., 2023; Shao et al., 2021; Wei et al., 2024). Spearman correlation analysis in our study also revealed a negative association between FTD and SE (*ρ* = −0.41, *P* < 0.001) and a positive association between FTD and AL (*ρ* = 0.39, *P* < 0.001). In contrast, the correlation between FTD and CR was weak and lacked clinical relevance, despite being statistically significant (*ρ* = 0.15, *P* = 0.013). This finding differed from that of Gong et al. (Gong et al., 2023). Their study found that a higher FT grade was independently associated with a larger CR, which may be attributed to the corneal developmental characteristics of their study population, mainly consisting of children aged 9–12 years with low myopia. The regression analysis further confirmed a significant cross-sectional association between FTD and SE after adjusting for age and sex (*P* < 0.001). Given the narrow range of FTD values in our sample, a “one-unit increase” lacks clinical meaning. Therefore, we rescaled the coefficient to a 0.01 increase in FTD, which was associated with a 0.07 D reduction in SE (β = −7.17 per 1.00 FTD, equivalent to −0.07 per 0.01 FTD, 95% CI: −0.10 to −0.05, *P* < 0.001). Age and sex were also independently associated with SE (both *P* < 0.001), consistent with known epidemiological patterns (Morgan et al., 2021). This finding underscores a strong quantitative relationship between elevated FTD and greater severity of myopia, although the cross-sectional design precludes any causal interpretation.

The ROC analysis for FTD yielded an AUC of 0.75 (95% CI: 0.68–0.81), with an optimal cutoff of 0.03 for identifying HM (sensitivity: 0.62, specificity: 0.76, PPV: 0.57, NPV: 0.80), indicating that FTD alone has moderate clinical utility as an independent confirmatory parameter. Our finding that FTD alone yields moderate discriminative accuracy for HM (AUC = 0.75) aligns with a recent adult occupational study (AUC = 0.78) (Xiao et al., 2026), suggesting consistent performance across age groups. Given the cross-sectional nature of this study, this cutoff of 0.03 should be interpreted as a preliminary reference for HM risk assessment in AI-based screening rather than a definitive diagnostic threshold. Prospective longitudinal studies are needed to validate its predictive value and generalizability to other populations. The significantly lower discriminative capacity of FTD compared to AL and AL/CR (both *P* < 0.05) can be partially explained by the more limited research on FTD, in contrast to the robust evidence and established biological link between AL, AL/CR and myopia (Bikbov et al., 2024; Liu et al., 2024; Tao et al., 2021). Given the cross-sectional design of this study, we cannot determine whether FTD changes precede or follow myopia progression. Therefore, future large-scale prospective studies are warranted to validate FTD’s utility, and, crucially, to assess whether changes in FTD occur prior to myopia progression using longitudinal data. Since combining multiple parameters enhances myopia detection performance (He et al., 2015; Liu et al., 2024; Zhao et al., 2022). We also evaluated the ROC curves for FTD combined with AL and AL/CR. When FTD was combined with AL/CR, the AUC increased to 0.94 (95% CI: 0.91–0.97), representing the highest diagnostic accuracy compared with all other variables (all *P* < 0.05). The combination of FTD with AL/CR model demonstrated a superior balance between sensitivity (0.90) and specificity (0.85), as reflected in its high Youden’s index of 0.75. Furthermore, the PPV and NPV of this combined model were 0.75 and 0.96, respectively, indicating excellent ability to correctly identify both true positives and true negatives. This makes it a robust tool for both screening and confirmatory diagnosis. Notably, the combined model significantly outperformed AL/CR alone (AUC: 0.94 vs. 0.91, *P* = 0.013), suggesting that FTD provides complementary structural information not fully captured by AL/CR alone. The exceptional performance of the combination of FTD and AL/CR suggests that FTD may provide complementary structural information not fully captured by AL alone. However, It should be noted that the AUC of 0.94 for the combined model was derived from the same sample used to build the model, without an independent external test set. Thus, the results should be interpreted as preliminary, and external validation in independent cohorts is warranted to confirm the generalizability of the combined model.

This study represents the first investigation to evaluate the applicability of AI-quantified FTD focusing on Chinese children with myopia, a critical target population for myopia control. Although our findings suggest that the performance of FTD as a standalone indicator for the identification of HM is limited, this study nevertheless holds clinical value by providing foundational evidence for the combined use of structural biomarkers in myopia management. Exploratorily, this methodology might serve as a possible adjunct or, in select scenarios, an alternative to cycloplegic refraction pending further validation and holds potential for future applications by enabling the identification of HM in large-scale population screenings, offering particular utility for patients with myopia who are unsuitable for cycloplegic examinations due to issues with poor cooperation, contraindications, or sensitivity to adverse effects (Foo et al., 2016; Wang et al., 2020; Wang et al., 2018).

Still, this study is constrained by several factors. First, the cross-sectional design limits the possibility of identifying a causal connection between FTD and HM. Second, this study was conducted at a single center (Beijing Children’s Hospital) and included only participants of a single ethnicity (Chinese Han). Consequently, the single-center, single-ethnicity design may limit the generalizability of our overall findings and conclusions to other ethnic groups or pediatric populations in different geographic regions within or outside China. Third, the hospital-based recruitment approach may introduce selection bias, as our sample likely overrepresents children seeking specialist care and may not be representative of community-based or general pediatric populations. Fourth, the sample size was limited, partly due to concerns about the safety and privacy of AI technologies. Fifth, the deep learning model’s performance is dependent on retinal image quality. In routine clinical practice, image variability (e.g., due to operator experience, patient cooperation, or device differences) may pose challenges to the robustness and reproducibility of the model, and real-world deployment would require rigorous quality control and standardization protocols. Sixth, our analyses did not account for several well-established confounders of myopia severity, including parental myopia history, outdoor activity time, and near-work exposure, as these data were not available in the current dataset. This omission represents a notable limitation, as these factors could influence the relationship between FTD and HM. To further elucidate the discriminative or even predictive capacity of FTD for HM, future investigations should prioritize comprehensive large-scale, multi-center, longitudinal studies encompassing diverse ethnic and socioeconomic populations, with systematic collection of key environmental and genetic confounders, as well as standardized image acquisition protocols.

In conclusion, AI-quantified FTD is strongly associated with the severity of myopia and demonstrates potential as a discriminative biomarker for HM. The combination of FTD and AL/CR significantly improves the discriminatory accuracy of HM identification, which may provide a novel, objective, and reproducible tool for large-scale screening and clinical management, pending validation in an independent external cohort.

## Data Availability

The raw data supporting the conclusions of this article will be made available by the authors, without undue reservation.
